# Gut microbiome populations are associated with structure-specific changes in white matter architecture

**DOI:** 10.1038/s41398-017-0022-5

**Published:** 2018-01-10

**Authors:** Irene M. Ong, Jose G. Gonzalez, Sean J. McIlwain, Emily A. Sawin, Andrew J. Schoen, Nagesh Adluru, Andrew L. Alexander, John-Paul J. Yu

**Affiliations:** 1 Department of Biostatistics and Medical Informatics, University of Wisconsin–Madison, Madison, WI 53705 USA; 2 Carbone Cancer Center, University of Wisconsin School of Medicine and Public Health, Madison, WI 53705 USA; 3 Department of Medical Physics, Wisconsin Institutes for Medical Research, University of Wisconsin School of Medicine and Public Health, Madison, WI 53705 USA; 4 Department of Radiology, University of Wisconsin School of Medicine and Public Health, E3/366 Clinical Science Center, 600 Highland Avenue, M/C 3252, Madison, WI 53792-3252 USA; 5 Department of Computer Sciences, University of Wisconsin–Madison, Madison, WI 53706 USA; 6 Waisman Laboratory for Brain Imaging and Behavior, University of Wisconsin–Madison, Madison, WI 53705 USA; 7 Department of Psychiatry, University of Wisconsin School of Medicine and Public Health, Madison, WI 53705 USA; 8 Department of Biomedical Engineering, College of Engineering, University of Wisconsin–Madison, Madison, WI 53706 USA; 9 Neuroscience Training Program, Wisconsin Institutes for Medical Research, University of Wisconsin–Madison, Madison, WI 53705 USA

## Abstract

Altered gut microbiome populations are associated with a broad range of neurodevelopmental disorders including autism spectrum disorder and mood disorders. In animal models, modulation of gut microbiome populations via dietary manipulation influences brain function and behavior and has been shown to ameliorate behavioral symptoms. With striking differences in microbiome-driven behavior, we explored whether these behavioral changes are also accompanied by corresponding changes in neural tissue microstructure. Utilizing diffusion tensor imaging, we identified global changes in white matter structural integrity occurring in a diet-dependent manner. Analysis of 16*S* ribosomal RNA sequencing of gut bacteria also showed changes in bacterial populations as a function of diet. Changes in brain structure were found to be associated with diet-dependent changes in gut microbiome populations using a machine learning classifier for quantitative assessment of the strength of microbiome-brain region associations. These associations allow us to further test our understanding of the gut-brain-microbiota axis by revealing possible links between altered and dysbiotic gut microbiome populations and changes in brain structure, highlighting the potential impact of diet and metagenomic effects in neuroimaging.

## Introduction

The tremendous diversity and abundance of microbes comprising the gut microbiome and its impact in numerous aspects of human health, physiology, and disease have emerged as an intense area of interest. Already, many important associations have been made that reveal the critical role the gut microbiome plays in inflammatory bowel disease, immunology, and host metabolism in both local and systemic disease^1^. The central nervous system (CNS) is no less susceptible with commensal bacteria able to shape complex social, emotional, and anxiety-like behaviors in both mice and humans^2,3^. Gut microbiota have also been shown to be integral to normal brain and behavioral development and in the regulation of the CNS and brain function^4^.

Disruptions in the number, composition, and taxonomic diversity of the gut microbiome have long been implicated in the development and exacerbation of neuropsychiatric conditions. Mood disorders are seen in more than half of all patients with irritable bowel syndrome^5^ and in a maternal immune-activation mouse model known to display features of autism spectrum disorder, treatment with *B. fragilis* ameliorated defects in anxiety-like and sensorimotor behaviors^2^. Diet-dependent microbiome populations are also strongly linked with psychopathological outcomes, as consumption of a high-fat diet is associated with reduced synaptic plasticity^6^ and low-calorie diets associated with augmented neurogenesis and brain-derived neurotropic factor (BDNF) levels, and improved cognition in adult rats^7^. More striking is the role of the gut microbiome in both prenatal and post natal neurodevelopment. Maternal high-fat diet-induced obesity in mice is associated with social behavioral deficits, which are mediated by alterations in the gut microbiome of the offspring^8^. The gut microbiome, which also critically promotes maintenance of microglia in the CNS during developmentally sensitive periods, is responsible for synaptic pruning and remodeling during development and adulthood^9^. Perturbations to the gut microbiome during these sensitive neurodevelopmental windows are associated with a wide range of neurodevelopmental and psychiatric disorders including anxiety, attention deficit hyperactivity disorder, depression, and schizophrenia^10^. Enigmatically, for all the evidence of the critical role that the gut microbiome serves during neurodevelopment and later in preserving CNS homeostasis, intestinal microbial communities are surprisingly sensitive and responsive to environmental cues such as diet, exposure to antimicrobials, and disrupted sleep patterns^10^. So dynamic are these communities that changes in diet are able to bring about rapid changes in the composition of the gut microbiome as fast as within a single day^11^. While strong associations between diet-dependent gut microbiome populations and neuropsychiatric illness are well recognized, it is unknown whether there exists an attendant structural neural correlate to parallel these observed changes in brain function and behavior. Given the striking spectrum of behavior observed as a function of diet, we hypothesized that diet-dependent changes in brain function and behavior also manifest with corresponding changes in neural tissue microstructure.

## Materials and methods

### Animals and experimental design

Animals were housed and cared for in an AAALAC-accredited facility and all animal experiments were conducted in accordance with University of Wisconsin-Madison IACUC-approved protocols. Following weaning, post natal day 22 male outbred Sprague-Dawley littermate rats (Charles River, Wilmington, MA, USA), were singly housed and randomized to one of four purified and irradiated diets: a control (chemically purified) diet (A), a high fat diet (B), a high fiber (C), and a high protein, low carbohydrate diet (D). Male animals were chosen to avoid potentially confounding estrous effects; male animals were also singly housed to additionally avoid the potentially confounding effects of rat coprohagy and its impact on gut microbiome populations. Animals began the experimental diets (*n* = 5 for each experimental diet) immediately post-weaning and were fed for a total of 21 days with free access to their assigned diet and water; all animals were maintained on a 12:12-h light–dark cycle. The high fat diets had 60% energy from fat sourced from combined lard and soybean oil, whereas the control diet had 17% energy from fat sourced solely by soybean oil. The high-fiber low energy diet was comprised of 40% fiber supplied by additional cellulose and provided 2.5 Kcal/g of diet relative to the 3.8 Kcal/g supplied by the control diet. The high protein low carbohydrate diet had 75% of its energy supplied by protein and 6% of energy derived from carbohydrate sources (Teklad, Madison, WI: TD97184, TD150669, TD150670, TD150671). Fecal samples from each of the immediately post-weaned animals were collected (prior to initiating their assigned diet) with a second animal-matched fecal sample subsequently collected from each animal three weeks later. All animals were then brought to a surgical plane of anesthesia and were transcardially perfused with ice-cold 4% paraformaldehyde (PFA). Brains were then cleanly dissected from the cranial vault, post-fixed in 4% PFA, and stored at 4 °C. Forty-eight hours prior to imaging, brains were serially washed in 1X PBS to minimize the attenuating effects of fixative prior to being placed in a custom-built holder filled with Fluorinert (FC-3283, 3 M) to minimize magnetic susceptibility artifact.

### Image data acquisition

For ex-vivo diffusion tensor imaging (DTI) acquisition, groups of 2 or 3 brains were simultaneously imaged using a 4.7-T Agilent MRI system and 3.5-cm diameter quadrature volume RF coil. Diffusion-weighted imaging (DWI) data were acquired employing a multi-slice spin echo sequence with the following imaging parameters: repetition time: 2000 ms; echo time: 24.17 ms; field of view of 32 × 32 mm with an acquisition matrix size 192 × 192 for a 0.17 × 0.17 mm in-plane resolution and 35 slices with 1 mm thickness. Diffusion measurements were acquired with diffusion encoded along 30 non-collinear directions at *b* = 1200 s /mm^2^ and three additional non-diffusion weighted (*b* = 0 s/mm^2^) measurements. The acquisition was averaged across two repeats resulting in a total imaging time of approximately 11 h.

#### Image preprocessing

Experimenters were blinded to the experimental conditions for this and all subsequent image processing steps. An affine registration tool^12^ from the FMRIB software library (FSL; http://fsl.fmrib.ox.ac.uk/fsl/fslwiki/) suite was used to co-register individual diffusion images to correct for subtle eddy current distortions. Gradient directions were then corrected for rotations^13^. Following these corrections, FSL was used to fit the diffusion tensors at each voxel.

#### Image spatial normalization

The FSL DTI output volumes were converted to NIfTI tensor format for compatibility with the DTI-TK^14^ software platform. Following the conversion, diffusivity units were verified to be in 10^−3^ mm^2^/s as required by the DTI-TK normalization routine. Utilization of a group-wise template has been shown to have a positive impact on population comparison analysis of diffusion markers such as TBSS^15^. Therefore, DTI-TK was employed to first estimate a study-specific tensor template from all imaging data sets acquired from the control group. This template was then used as a target to which each subject tensor volume was spatially normalized using the DTI-TK tensor-based registration tools. DTI-TK’s registration routine, which iteratively employs rigid-body, affine, and diffeomorphic transformations, was used as it has been shown to offer improved registration results over other registration algorithms^16^.

#### Image tract-based spatial statistics

The TBSS formalism was implemented as recommended in Bach et al.^17^ This consists of replacing the traditional TBSS registration (FSL’s FNIRT) with the DTI-TK registration routine, which uses the full tensor information for the registration resulting in an improved alignment quality. The rest of the TBSS pipeline was applied utilizing the recommended parameters implemented in FSL, which includes using a 0.2 FA threshold for creating the white matter skeleton. Additionally, a permutation test with *n* = 252 corrected for multiple comparisons and threshold-free cluster enhancement (TFCE)^18^ was implemented with FSL’s Randomize for inter-group comparisons (i.e., three comparisons, each with *n* = 10), with *p* < 0.05 as threshold for significance.

#### Image regions of interest (ROI) analysis

A standard atlas^19^ from which anatomical ROIs were derived was non-linearly aligned to the group-wise template. The registration included elastic warping with a point-set based registration metric implemented in the advanced normalization tools (ANTS)^20^ software library. For use in the subsequent statistical analysis, mean values of the diffusion-tensor-derived indices, namely, fractional anisotropy (FA), RD (radial diffusivity), AD (axial diffusivity), and TR (trace: mean diffusivity [MD] × 3) were computed within each ROI for all individual samples. These values were computed in the group-wise template space.

### Construction and sequencing of V3/V4 16*S* metagenomic libraries

Fecal matter was collected using Oragene OMNIgene-Gut storage tubes (DNA Genotek, Ottawa, ON, Canada) and DNA was isolated from 250 uL of fecal slurry using the OMNIgene adapted MO BIO PowerFecal DNA Isolation Kit (Mo Bio Laboratories Inc, Carlsbad, California, USA) at the University of Wisconsin-Madison Biotechnology Center. DNA concentration was verified using the Qubit® dsDNA HS Assay Kit (Life Technologies, Carlsbad, California, USA). Samples were prepared as described in the 16 *S* Metagenomic Sequencing Library Preparation Protocol, Part # 15044223 Rev. B (Illumina Inc., San Diego, California, USA) with the following modifications: The 16 S rRNA gene V3/V4 variable region was amplified with nested primers (forward primer: 5′-ACACTCTTTCCCTACACGACGCTCTTCCGATCTCCTACGGGNGGCWGCAG-3′, reverse primer: 5′-GTGACTGGAGTTCAGACGTGTGCTCTTCCGATCTGACTACHVGGGTATCTAATCC-3′), Region-specific primers were previously described in Klindworth et al.,^21^ and were modified to add Illumina adapter overhang nucleotide sequences to the gene-specific sequences. Following initial amplification, library size was verified on an Agilent DNA1000 chip, and cleaned using a 1x volume of AxyPrep Mag PCR clean-up beads (Axygen Biosciences, Union City, CA). Illumina dual indexes and Sequencing adapters were added using the following primers (Forward primer: 5′-AATGATACGGCGACCACCGAGATCTACAC[55555555]ACACTCTTTCCCTACACGACGCTCTTCCGATCT-3′, Reverse Primer: 5′-CAAGCAGAAGACGGCATACGAGAT[77777777]GTGACTGGAGTTCAGACGTGTGCTCTTCCGATCT -3′, where bracketed sequences are equivalent to the Illumina Dual Index adapters D501-D508 and D701-D712). Following PCR, DNA was cleaned using a 1x volume of AxyPrep Mag PCR clean-up beads (Axygen Biosciences). Quality and quantity of the finished libraries were assessed using an Agilent DNA1000 chip and Qubit® dsDNA HS Assay Kit, respectively. Libraries were standardized to 2 μM and pooled prior to sequencing. Paired end, 300 bp sequencing was performed using the Illumina MiSeq Sequencer and a MiSeq 600 bp (v3) sequencing cartridge. Images were analyzed using the standard Illumina Pipeline, version 1.8.2.

### 16*S* rRNA sequence preprocessing and analysis

The University of Wisconsin-Madison Biotechnology Center performed data analysis on the 16*S* rRNA sequencing. Briefly, sequencing reads were adapter and quality trimmed using the Skewer^22^ trimming program. Flash^23^ was used to merge paired end reads into amplicon sequences. Merged amplicons were then quality filtered. Qiime^24^ analysis used an open-reference operational taxonomic unit (OTU) picking process: reads are clustered against a reference sequence collection using 97% similarity, and any reads which do not hit the reference sequence collection are subsequently clustered de novo. Alignments were filtered to remove variable regions prior to creating the phylogenetic tree. Singleton OTUs and OTUs that could not be aligned using PyNAST were removed. Alpha diversity was calculated with a rarefaction upper limit of (median depth/sample count). Beta diversity was leveled according to the lowest sample depth.

### Microbiome and brain random forest analysis

*R* was used for the preprocessing and downstream predictive analysis of the metagenome and brain measurement/regions^25^. The relative abundance values of the metagenome OTUs were transformed using log (base 2). The brain measurement/regions were then *z*-scaled across the samples. A model was built to predict the diet for each data set separately using the Random Forest package^26^ in *R* (ntree = 16,000). The prediction performance and confusion matrices were determined using leave-one-out cross-validation. The percent mean decrease accuracy from the importance matrix was used to select features that were most predictive of diet. Heat maps were generated using the *R* package. Features from the quantitative microbiome profiles including species-level relative abundances were mapped to the brain imaging data from the same subjects. Next, we predicted each *z*-scaled brain region using Random Forest in regression mode (ntree = 4000) with metagenome OTUs as input. Feature selection was done using %IncMSE as calculated by the importance matrix.

To find OTU and brain region pairs that are individually predictive of diet and whose brain measurement is predictable by the OTU, we derived four different scores with values from 1/*N*_element_ (lowest, least importance) to 1 (highest, most important). We derive scores from the metagenome (*S*(OTU_i_)) and brain measurement/regions (*S*(BrainM_j_)) from the rank of MeanDecreaseAccuracy from the random forest importance matrix when predicting diet. For each brain measurement/region and metagenome OTU, we derive a score based upon the rank of the OTU’s %IncMSE for predicting the brain region/measurement. The multiplication of the *S*(OTU_i_), *S*(BrainM_j_), and *S*(OTU_i_,BrainM_j_) derives the combined score (*S*_Combined_(OTU_i_,BrainM_j_).

$$S\left( {OTU_i} \right) = \frac{{Rank\left( {MeanDecreaseAccuracy\left( {Diet\left| {OTU_i} \right.} \right)} \right)}}{{N_{OTU}}}$$

$$\left ( {BrainM_j} \right) = \frac{{Rank\left( {MeanDecreaseAccuracy\left( {Diet\left| {BrainM_j} \right.} \right)} \right)}}{{N_{BrainM}}}$$

$$S\left( {OTU_i,BrainM_j} \right) = \frac{{Rank\left( {{\mathrm{\% }}IncMSE\left( {BrainM_j\left| {OTU_i} \right.} \right)} \right)}}{{N_{OTU}}}$$

$$S_{Combined}\left( {OTU_i,BrainM_j} \right) = S\left( {OTU_i} \right)S\left( {BrainM_j} \right)S\left( {O{\mathrm{T}}U_i,BrainM_j} \right)$$

After ordering all OTU, Brain region/measurement pairs using *S*_combined_, we then selected the top 27 ranked pairs for building three Random Forest models, two using either OTU and Brain region/measurement for predicting diet, and one using both the OTUs and Brain region/measurement for predicting diet. A Circos plot was generated using circlize^27^.

## Results

### Diet modulates white matter structural integrity

**Fig. 1:**
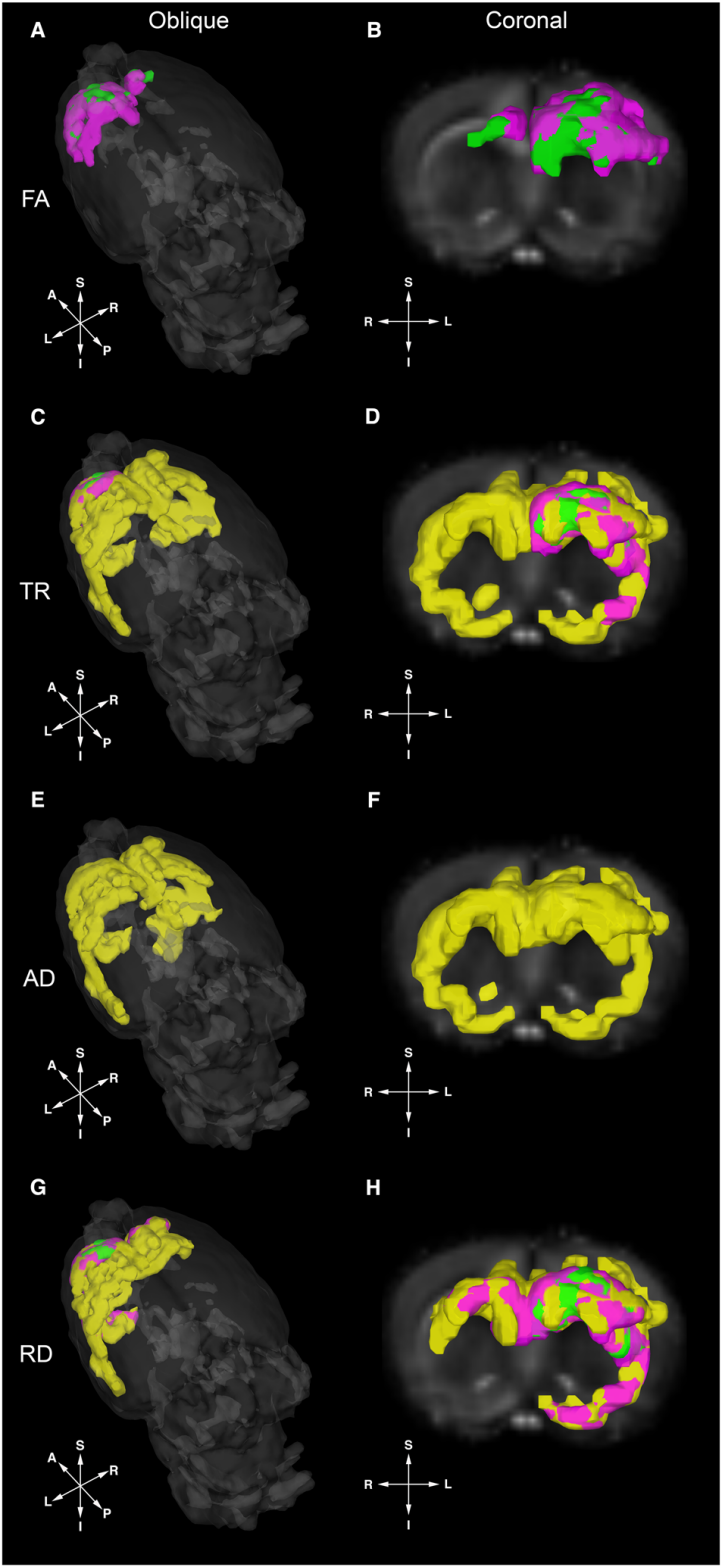
Diet impacts multiple measures of the diffusion tensor in a diet-dependent manner. Ex-vivo diffusion tensor imaging (DTI) was performed 21 days after immediately post-weaned male rats were assigned to one of four assigned diets (*n* = 20; *n* = 5 in each diet group) to measure diet-dependent changes in the diffusion tensor when referenced to control animals. Following tract-based spatial statistics (TBSS) analysis, areas of increased FA (**a**, **b**) and decreased trace (TR) (**c**, **d**) axial (AD) (**e**, **f**) and radial diffusivity (RD) (**g**, **h**) for each experimental diet group (as compared to the standard diet) were identified and are displayed over masked oblique and coronal fractional anisotropy (FA) maps. No voxels corresponding to decreased FA or increased TR, AD, or RD were identified. Areas highlighted in yellow (high fat), green (high fiber), and magenta (high protein, low carbohydrate) represent diet-specific voxels where tract-based spatial statistics (TBSS) analysis revealed statistically significant diffusion tensor differences between each of the enriched diet groups and the control diet group. No significant changes were found in FA for the fat diet group or in AD for the fiber diet group

To explore the influence of diet on neural tissue microstructure and organization, singly housed, immediately post-weaned male rats were randomized to one of four purified and irradiated diets: a standard/control diet (A), a high fat diet (B), a high fiber diet (C), and a high protein, low carbohydrate diet (D). After three weeks on their assigned diet, ex-vivo whole brain DTI was performed. After data preprocessing and spatial normalization, the imaging data were analyzed with tract-based spatial statistics (TBSS). Analysis with TBSS revealed confluent areas of statistically significant differences in FA (*p* < 0.05, corrected for multiple comparisons) between the control diet group and both the fiber and protein diet groups with areas of increased FA clustering in the left frontal neocortex of both the fiber diet group and in the protein diet group (Fig. 1a, b). While no significant differences in FA were identified between the standard diet and high-fat diet groups, there were extensive areas of decreased RD, AD, and TR (trace = mean diffusivity [MD] × 3) between the control and high-fat diet groups (*p* < 0.05, corrected for multiple comparisons, Fig. 1c–h). These areas of significant change include voxels spanning areas of the neocortex, the corpus callosum, the forebrain, and the right external and left internal capsules. The protein diet group also displayed significant reductions in RD and TR the left frontal neocortex with additional reductions in RD and TR in clusters encompassing voxels located in the expected regions of the left external capsule, left frontal neocortex, and the forebrain (Fig. 1d, h).

### Discriminatory OTUs and diffusion tensor regions of interest can predict diet

**Fig. 2:**
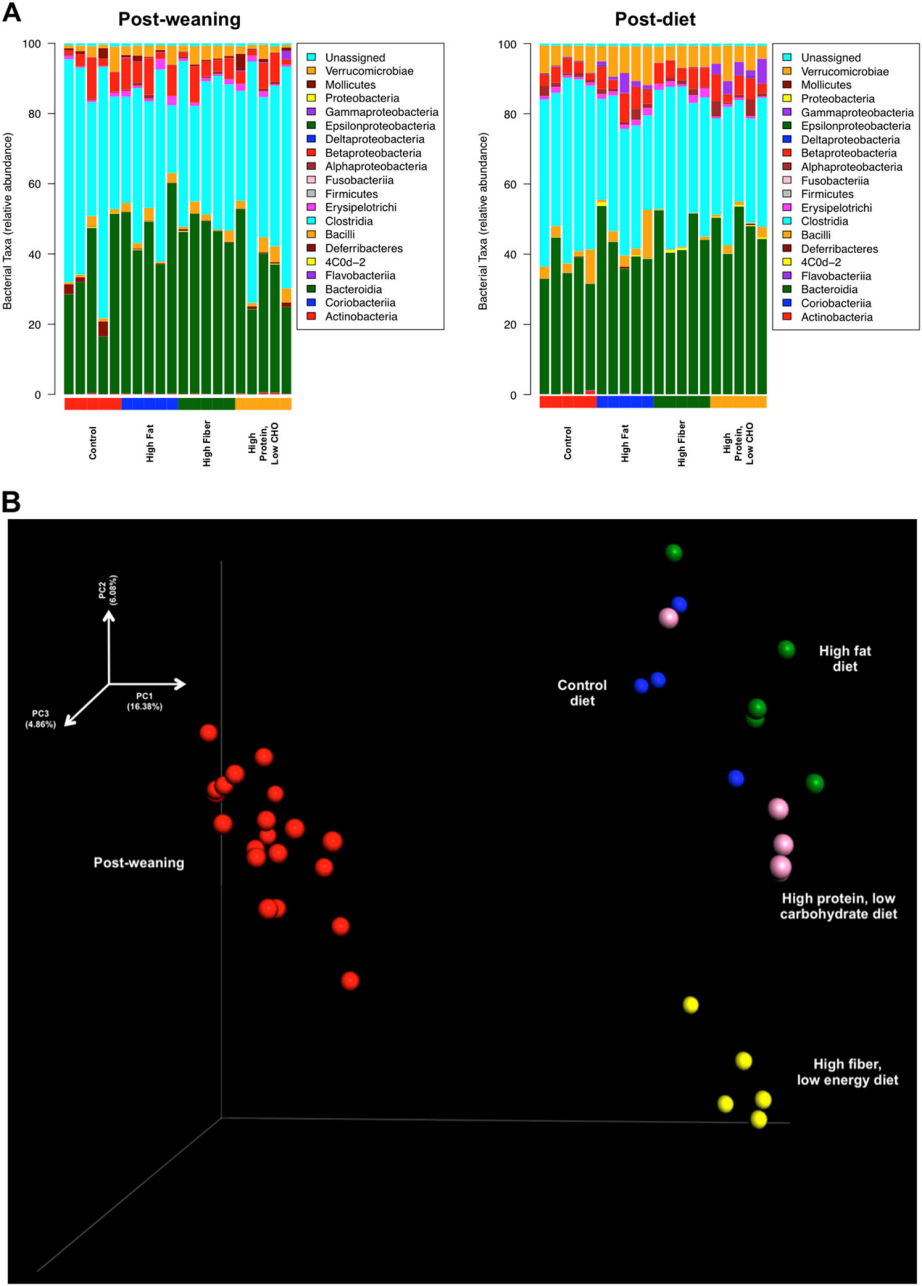
Diet shapes both the relative abundance and composition of gut microbiome populations. Immediately post-weaned male rats were singly housed and were assigned to one of four experimental diets for 21 days (*n* = 20) including a control (standard) chow, high fat, high fiber, and high protein, low carbohydrate diet. **a** Taxonomic distribution of major identified bacteria taxa (at the class level) immediately post-weaning and after 21 days on the assigned experimental diet with values representing the average relative abundance across all samples within the indicated group. **b** 16 *S* rRNA gene surveys (analyzed by weighted UniFrac-based PCoA) from immediately post-weaned animals and after 3-weeks on a control (blue), high-fat diet (green), high protein, low carbohydrate (pink), and high-fiber diet (yellow). Principle coordinates 1 and 2 (PC1, PC2) are the *x*- and *y-*axis, respectively, and are scaled on the basis of percent variance with PC3 depicted by the shading of each point

The constellation of these imaging findings reveals remarkable changes in underlying white matter structural integrity occurring in a diet-dependent fashion. As different diets also alter the composition of the gut microbiome, we next explored whether diet-dependent gut microbiome populations could be mediating these observed diet-specific perturbations in white matter structural integrity. To explore this hypothesis, fecal samples from each of the immediately post-weaned animals were collected (prior to initiating their assigned diet) with a second animal-matched fecal sample subsequently collected 21 days later. 16*S* ribosomal RNA sequencing was then performed on all fecal samples with amplification of the V3/V4 variable regions. A comparison of the composition of gut microbial communities across the different diets was assessed with the UniFrac metric^28^ and, as expected, significant changes were seen in the relative abundance of bacterial taxa at the end of the three week diet period (Fig. 2a). Principle coordinates analysis (PCoA) of weighted UniFrac-based pairwise comparisons (post-weaning; after diet) similarly revealed diet-specific gut microbiome communities that were unique to the consumed diet (Fig. 2b)^29^.

**Fig. 3:**
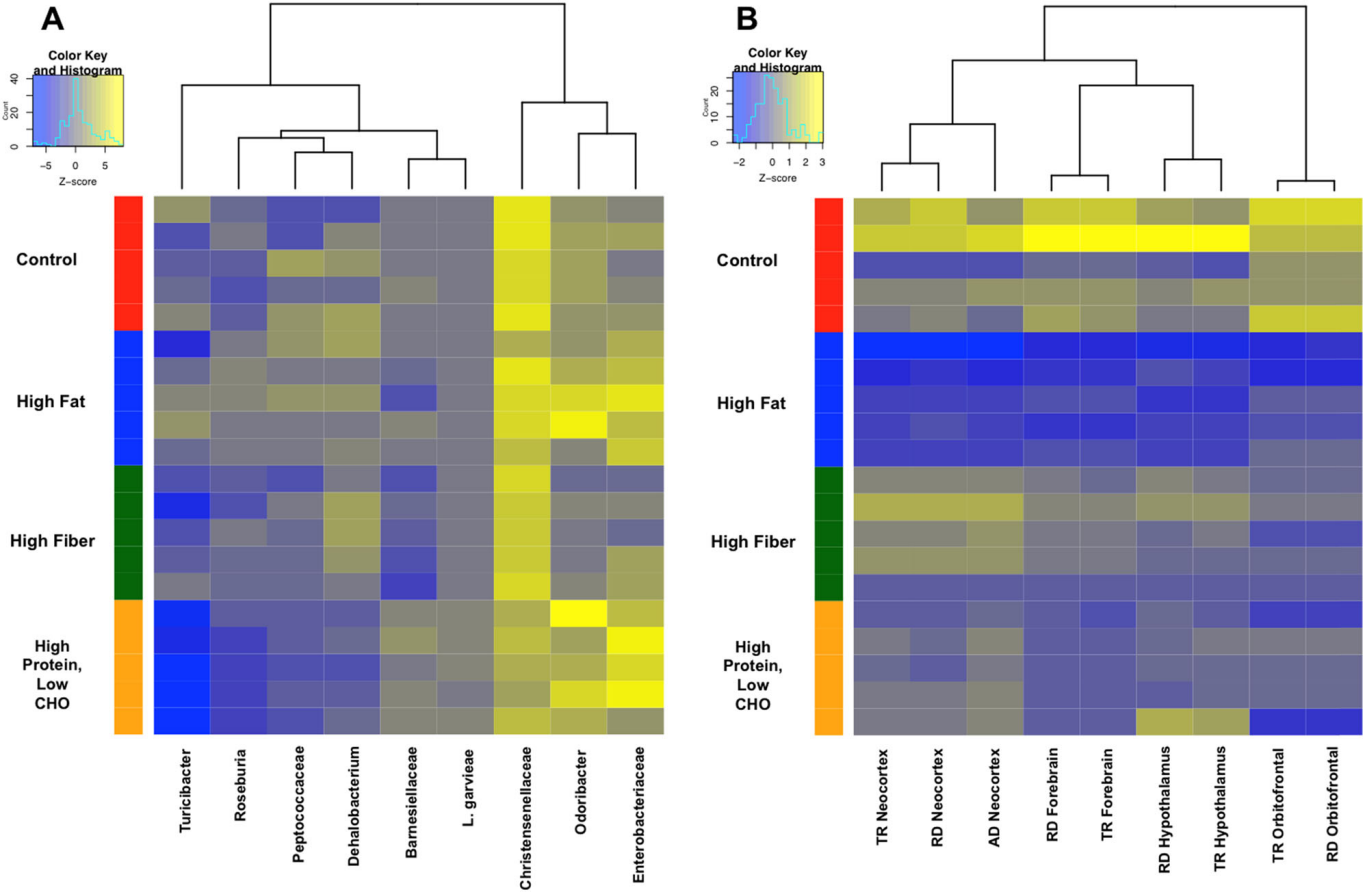
Discriminatory OTUs and measures of the diffusion tensor can both predict animal diet. Predictive modeling with Random Forest with leave-one-out cross-validation identified nine features from both the gut microbiome and from experimentally measured diffusion tensor values that were able to predict the diet of the animal with 100% and 95% accuracy, respectively. Hierarchically clustered heatmaps for discriminatory elements and their relationship to diet are shown here for identified OTUs (**a**) and for identified diffusion tensor measurements (**b**)

To refine the relationship between gut microbial communities, diet, and changes in neural tissue microstructure, predictive modeling with Random Forest (which can be thought of as an ensemble of decision trees) and leave-one-out cross-validation was performed^25,30^. We found that the log (base 2) relative abundance of the OTUs of gut microbiota between the first and second fecal samples were able to discriminate between the different diets with 100% accuracy using just nine OTUs (Fig. 3a; Table S1) where OTUs with the highest mean decrease accuracy (a feature importance metric from Random Forest) were used to select the features that were most important in determining the diet. To estimate the predictive accuracy on future data, we performed 3 × 5-fold cross-validation (stratified by diet) on the whole process of feature selection using the random forest ranking and achieve an average accuracy of 75 ± 10% (95% confidence interval assuming normal distribution with sample standard deviation; a random classifier would achieve 25% accuracy). We also determined the most predictive OTUs for each diet using Random Forest; in the case of the high protein, low carbohydrate diet, we found that the bacterial species *L. garvieae* could alone predict this diet (Fig. S1). In addition, Roseburia, and Barnesiellaceae were also selected as discriminative OTUs that are good predictors of the high-fiber diet (Fig. S1). With the identification of highly discriminatory and predictive elements in the OTU sequencing data, we next explored whether such elements were also present in our diffusion tensor data. Following TBSS processing, anatomical regions of interest (ROIs) derived from a standard atlas^19^ were aligned to a group-wise template, which was estimated from the samples in the standard diet group. Mean values of FA, RD, AD, and TR were then computed within each ROI for all individual samples. These mean values of diffusion-tensor-derived indices per ROI were used as an input to build a model to predict the diet for each data set separately. Again, using Random Forest with leave-one-out cross-validation, we identified nine features (nine imaging ROIs), which were able to successfully discriminate between the different diets with 95% accuracy (Fig. 3b; Tables S2-S4). Estimating the predictive performance on future ROI data using 3 × 5-fold cross-validation (stratified by diet), we achieve an average accuracy of 77 ± 12%.

### Diet-specific gut microbiota populations potentially influence white matter integrity

**Fig. 4:**
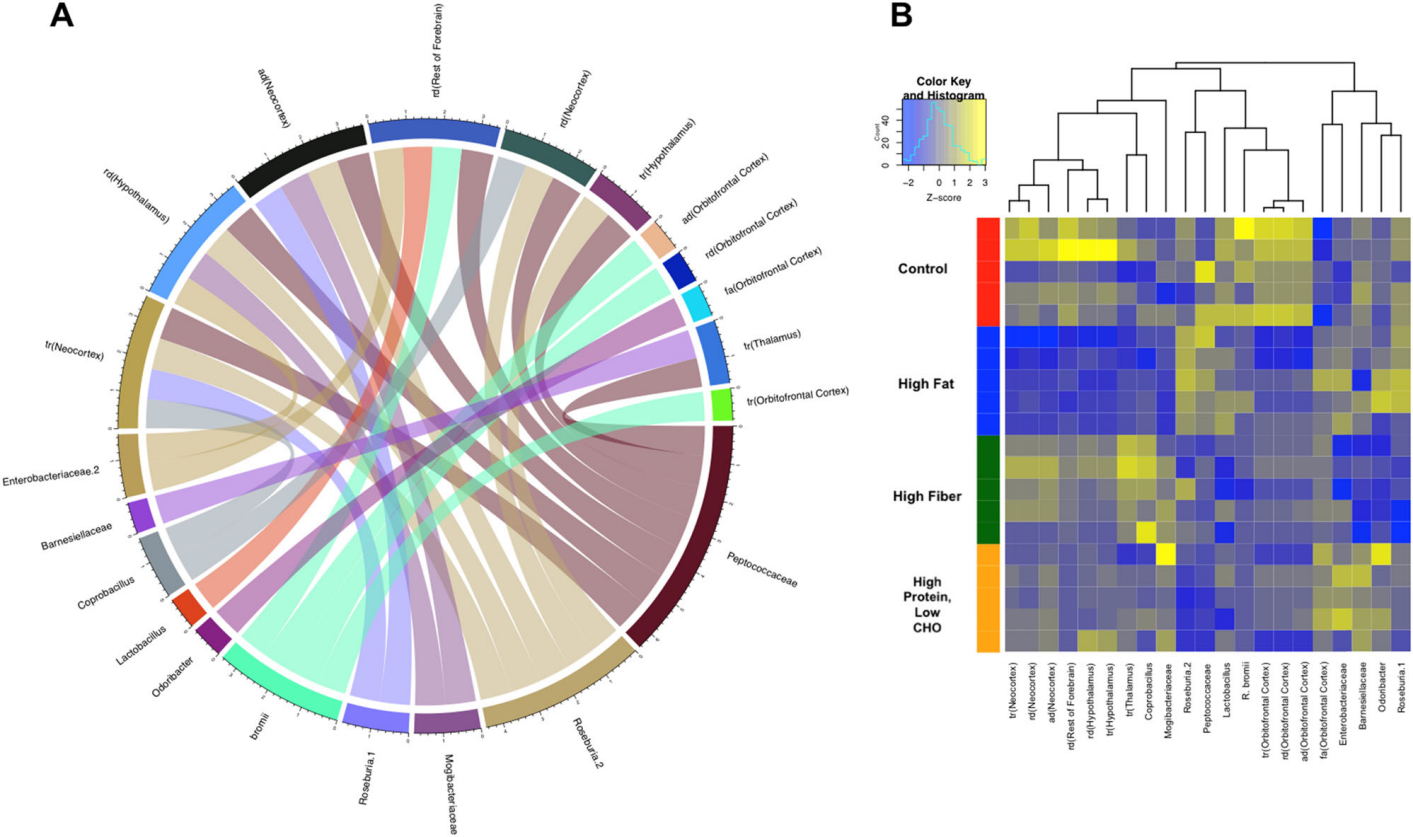
Specific bacterial populations are predictive of diffusion tensor measurements in corresponding brain ROIs. Regression-based ensemble analysis of OTUs and calculated diffusion tensor measurements identified unique populations of gut bacterial genera that are predictive of ROI-specific tensor changes (Table S5). A Circos plot (**a**) and a hierarchically clustered heatmap (**b**) illustrate the most robust predictive relationships between OTUs and brain diffusion tensor ROIs identified in our analysis. fa fractional anisotropy, tr trace, rd radial diffusivity, ad axial diffusivity

Beyond using diffusion tensor ROIs and OTUs to predict animal diet, we sought to extend our analysis to further characterize potential relationships between brain structure and populations of bacterial genera in the gut microbiome. Specifically, we sought to determine if individual OTUs could predict diffusion tensor measures (such as FA, RD, MD, or TR) in a brain ROI. To these ends, we predicted each *z*-scaled brain ROI using Random Forest in regression mode. Feature selection was then performed using percent increase mean squared error (MSE) as calculated by the importance matrix. The importance matrix demonstrates the degree to which MSE increases when a variable is randomly permutated; if a variable is randomly permutated, it will not contribute to accuracy in prediction and predictions therefore will not fluctuate. Conversely, important variables will significantly change the predictions if they are randomly permutated. To find OTU:brain ROI pairs that are individually predictive of diet and whose ROI value is predictable by the OTU, we derived a scoring function that combines rankings from three different ranked lists such that our constraints are met; we select for OTU features that are predictive of brain measurements and combined OTU features and brain measurements that are also predictive of diet. After ordering all OTU:brain ROI pairs using *S*_combined_, we then selected and used the top 27 ranked pairs for building three Random Forest models, two using either OTU or brain ROIs for predicting diet, and one using both the OTUs and brain ROIs for predicting diet (Table S5). Forward selection was then performed until we obtained 100% accuracy. With a selected threshold of 0.84, we were able to accurately identify OTU:brain ROI pairs that matched the presence of a gut microbiome OTU to a predicted and experimentally observed diffusion tensor value in a prescribed ROI (Fig. 4) thus demonstrating that diet-dependent gut microbiome populations can potentially predict or be linked to changes in white matter structural integrity in a defined manner.

## Discussion

The mechanism through which the gut microbiome exerts its effects on the CNS is multifactorial (neural, endocrine, and immunologic) but is thought to largely occur via the generation of bacterial metabolites, which exert their physiologic effects both locally and systemically. Short-chain fatty acids, produced by the bacterial fermentation of dietary carbohydrates, alter neuronal excitability^31^ and gut bacteria also manufacture a wide spectrum of neuroactive compounds that include dopamine, γ-aminobutyric acid, histamine, acetycholine and tryptophan, a precursor in the biosynthesis of serotonin^32^. The results of our work extend and build upon these findings by uncovering changes in white matter structural integrity and how they may be linked to specific gut microbiome populations. Other groups have previously linked the gut microbiome to structural changes in the brain^33^, and used machine learning methods to associate the gut microbiome with phenotypic data^34^; however, to the best of our knowledge, we are the first to utilize machine learning methods to directly link diet with gut microbiome populations and brain structure. Our findings associating gut microbiome populations to changes in brain structure are further buttressed with new evidence demonstrating the gut microbiome and Toll-like receptor 4 (TLR4) as critical stimulants of cerebral cavernous malformations^35^. Our method for uncovering potential links between gut microbiome populations and brain structural changes can help guide important new experiments to study how these microbiome populations impact the CNS beyond transiently modulating the presence and flux of neuroactive molecules and compounds.

Our observed structural changes may also be explained in part by recent studies demonstrating examples of how gut microbiome populations influence the transcriptional activity of genes involved in neuronal myelination^36,37^, which could potentially impart a lasting structural change and durable imprint on brain structure, function, and behavior. Intriguingly, all areas of significant FA change were the result of increased FA with no significant areas of decreased FA identified. While the cellular mechanisms for increases in FA remain unknown, increased water content in the myelin sheath, accelerated myelination and/or microscopic deficits of axonal structures or decreases in axonal diameter, packing density, and branching may all contribute to areas of elevated FA found in our study^38,39^. In addition, while there was an absence of significant FA differences between our high fat and standard diet groups, there were, however, substantial areas of significantly lowered AD, RD, and TR between these two groups (Fig. 1). While it is generally unexpected to observe an apparent decoupling in the direction of change in FA and AD as changes in either diffusion metric tends to follow the other, our results are not singularly unique as numerous prior reports have also made this observation^40–42^. With complex fiber architecture and subsequent orientation uncertainty, the direction of the measured tensor eigenvalues does not always correspond to the underlying structure, especially in instances where tensor measurement are being made in pathological tissue^40,41^. Additionally, different tensor shapes can yield a similar FA and the high degree of overlap between regions of concomitant reduction in both AD and RD may help explain the absence of FA differences in those regions between these two groups^43^. These results also highlight that even in the absence of a significant shift in FA, there are important changes occurring to the diffusion tensor that likely reflect important biological transformations.

In an extension of our work, we also performed a regression-based ensemble analysis of OTUs identified from sequenced 16*S* ribosomal RNA with calculated diffusion tensor measurements. From this analysis, we were able to identify unique populations of gut bacterial genera that were both associated with and predictive of ROI-specific tensor changes (Fig. 4). These associations were found to be independent of diet, which suggests that while the overall taxonomic composition and relative abundance of any one specific bacterial genera is diet-dependent, its contribution to underlying brain structure is not likely the result of synergistic effects derived from the presence of other bacterial populations or to behavioral changes that may result from a change in diet. This parallels the findings of numerous reports in the literature where the recolonization of a single gut bacterial species has been shown to be able to rescue perturbations in host physiology and ameliorate behavioral phenotypes^2,8^. With our Random Forest analysis not only predicting microbial driven ROI-specific changes but also changes to regions of the brain central to animal behavior including the neocortex, hypothalamus, and forebrain, these findings also highlight a potential mechanism whereby certain microorganisms are able to exert their systems-level behavioral effects. Notably, these predictions are again consistent with prior reports in the literature, with many of the bacterial genera identified in our analysis having been previously identified not only as important modulators of behavior but also having been shown to exert their effects in our predicted ROIs. A standout example of the predictive efficacy of our analysis is our identification of Roseburia being linked to microstructural changes in the neocortex (Fig. 4a). Previous reports have linked gut populations of Roseburia with elevated mood^44^, and with recent experimental findings implicating neocortical regions with altered mood and other affective disorders^45^, our analysis uncovers a potential hypothesis for how Roseburia is able to exert its specific behavioral effects. In sum, the methodology and results presented here are a novel framework with which one could potentially infer what brain structure may be given knowledge of the gut microbiome. In particular, our novel neuroimaging and machine learning classifier for the quantitative assessment of microbiome-brain region associations can guide future experimental work, whereby with an interest in a particular brain region or structure, our analysis can now allow for the selection of specific bacterial genera to generate a more tailored study of how specific gut microbiome populations impact brain structure, function, and behavior.

Our findings also bolster an emerging appreciation of metagenomic effects in experimental science. Particularly in experiments where mice and rats serve as a model organism, the absence of strong controls for metagenomic populations (such as the gut microbiome) may inadvertently confound experimental reproducibility as there are many potential linkages of the microbiome to variables known to influence experimental outcomes^46^. Our results now foster important experimental considerations for the neuroscience and neuroimaging community. Diffusion tensor imaging and the subsequent evaluation of white matter integrity are often employed in an effort to identify imaging endophenotypes across a broad range of neurologic and psychiatric diseases and the sensitivity of diffusion tensor imaging, coupled with its bias-free automated analysis, makes this an established and widespread clinical and experimental technique. Diffusion tensor techniques also serve as the basis for several large ongoing neuroimaging trials including the Alzheimer’s Disease Neuroimaging Initiative, the Human Connectome Project, and ENIGMA-DTI. With many clinical and experimental studies leveraging the utility and sensitivity of diffusion tensor imaging, that the gut microbiome could impart an unexpected impact on sensitive measures of diffusion tensor is an unanticipated and surprising challenge. While the effects of the gut microbiome are, in all likelihood, less likely to impact large well-organized white matter tracts such as the corpus callosum, studies employing DTI to identify novel imaging endophenotypes may be unknowingly affected by metagenomic effects that can further challenge efforts to identify and refine candidate neuroimaging biomarkers across a wide variety of diseases. This may be especially true for neuroimaging trials employing diffusion tensor techniques in schizophrenia, major depressive disorder, and bipolar disorder where investigators have yet to validate a truly robust neuroimaging biomarker that may have been influenced by unaccounted gut microbiome populations and their influence on the measurement of sensitive diffusion tensor indices.

In addition, concerns regarding the reproducibility of neuroimaging biomarkers have been raised with newly available data demonstrating the surprisingly poor reproducibility of candidate gene effects on imaging measures of mental illness. In an effort to countervail these findings, large imaging trials such as ENIGMA have pursued large sample sizes and meta-analyses as a means to screen for these false positive findings^47^; however, it is conceivable that these efforts could still be encumbered by the unaccounted contributions of gut microbiome populations on diffusion tensor metrics, thus masking and precluding the discovery of biologically important tensor changes and potentially salient neuroimaging biomarkers. These unforeseen potential challenges highlight a need to better understand the mechanism through which various bacterial genera are able to exert changes on brain structure and moreover, to uncover the mechanism by which they are able to do so in such a region-specific manner. These efforts will likely complement additional work exploring both the durability and malleability of these tensor changes and how these can be shaped not only by diet but also through the consumption of prebiotics, probiotics, and other means of dietary supplementation.

## Electronic supplementary material

Supplemental Material
