# Supplementary material for: Gut microbiome populations are associated with structure-specific changes in white matter architecture
Source: Transl Psychiatry. 2018 Jan 10;8:6. doi: 10.1038/s41398-017-0022-5 (PMC5802560; doi:10.1038/s41398-017-0022-5)
Supplement: Supplementary file 1 — Supplemental Material [file 41398_2017_22_MOESM1_ESM.docx]

**Gut microbiome populations are associated with structure-specific changes in**

**white matter architecture**

**Supplementary Materials**

Figure S1

Tables S1-S5


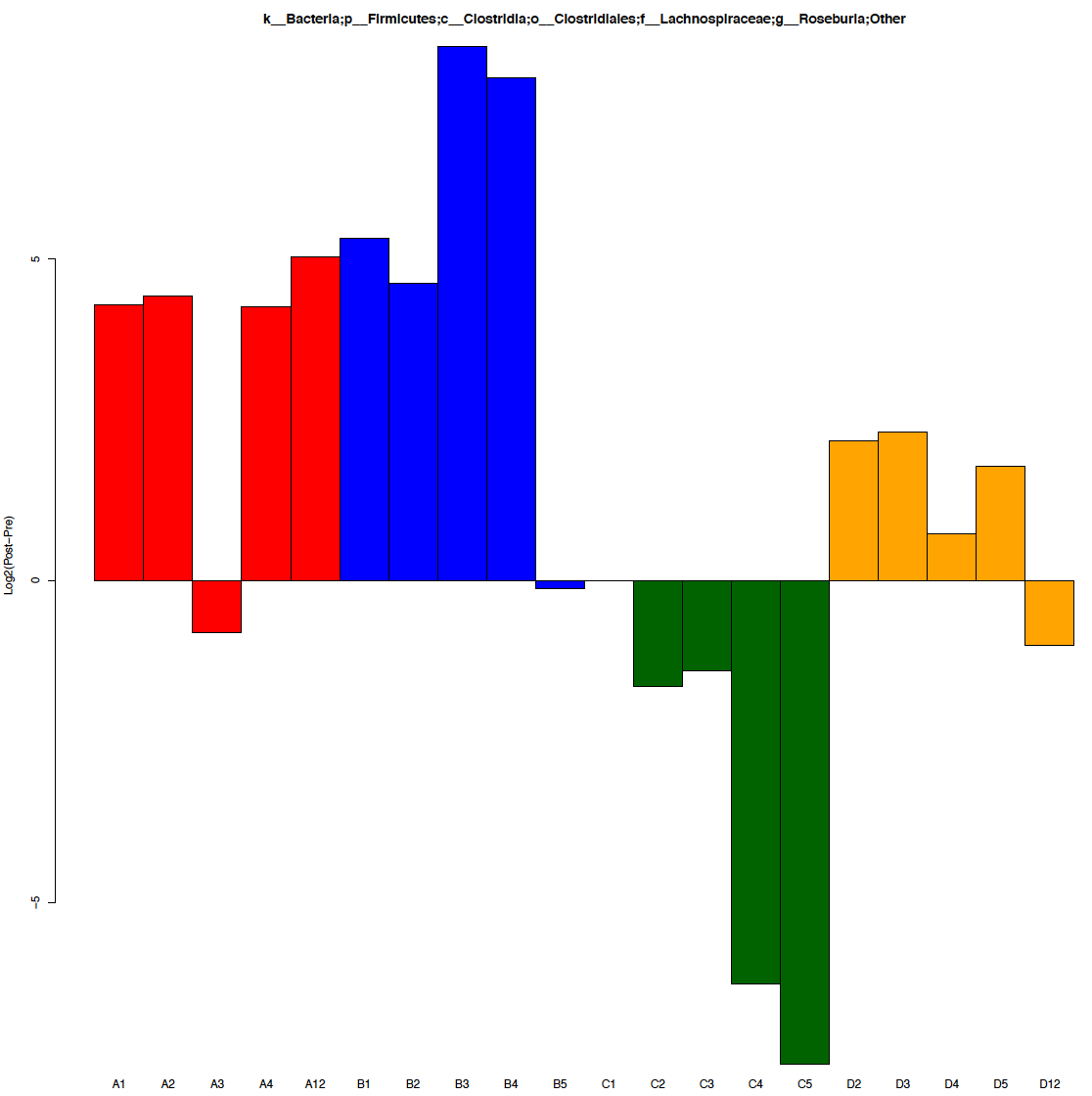

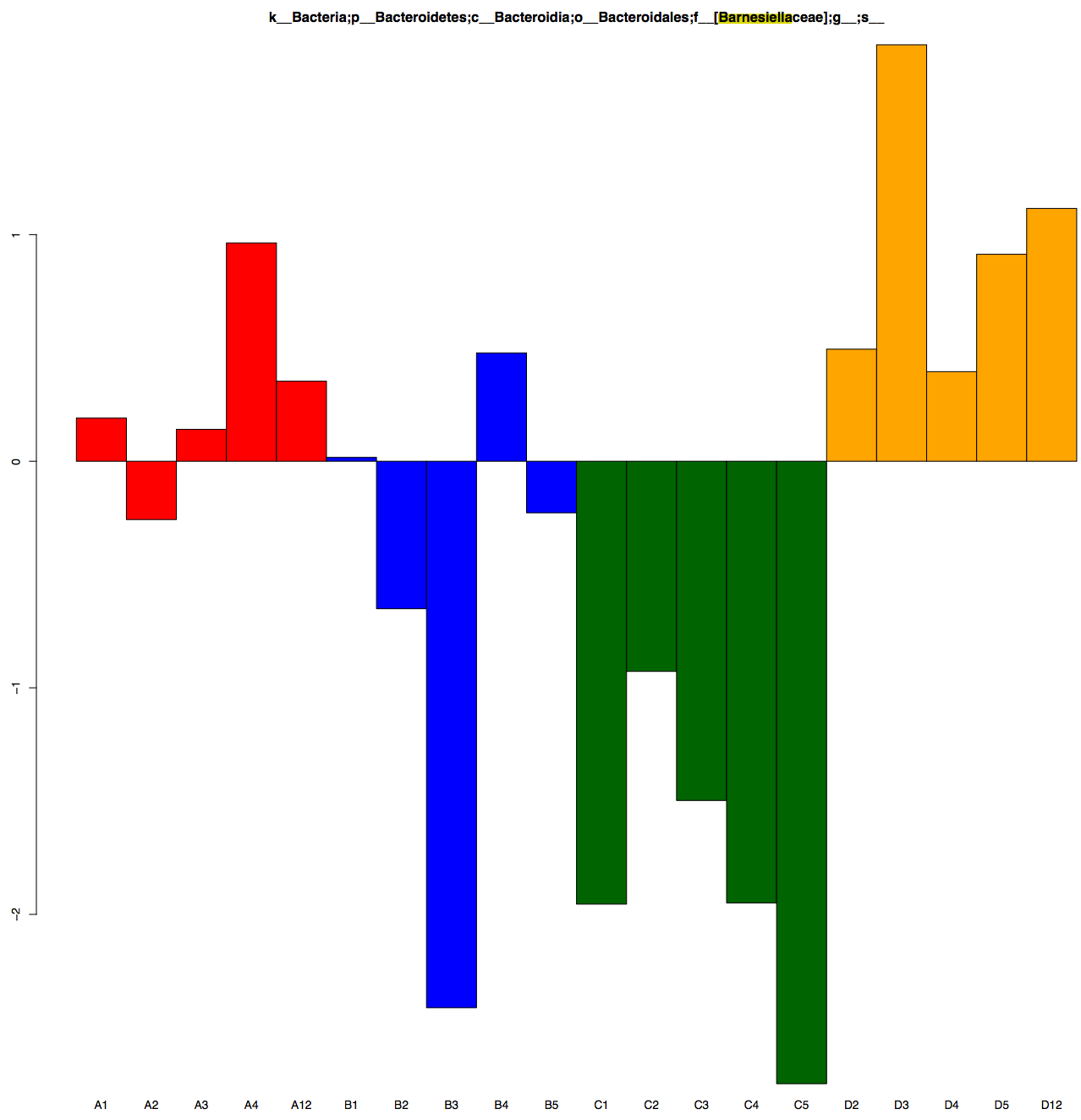

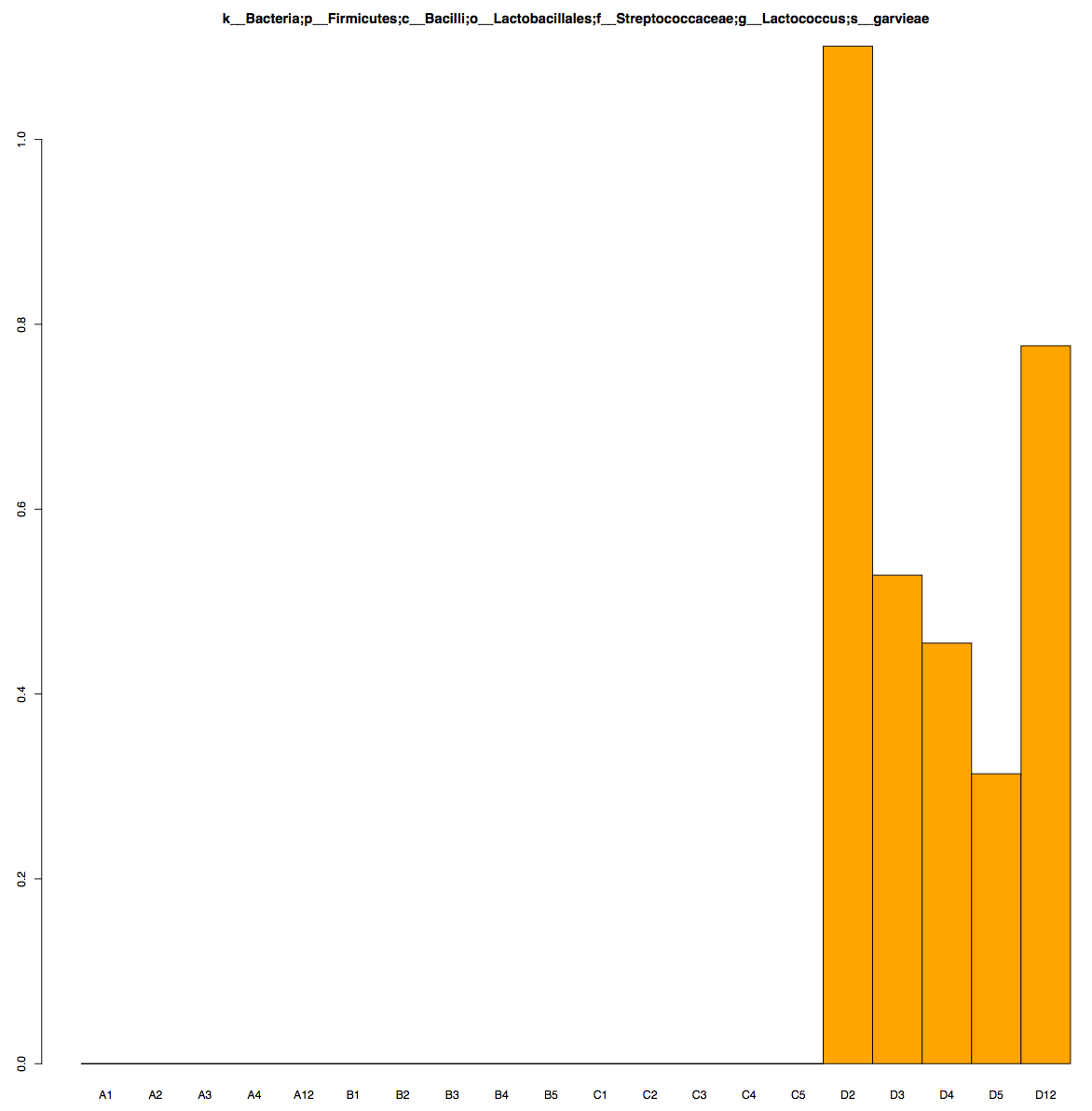


**A**

**B**

**C**


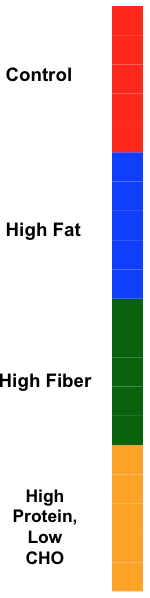

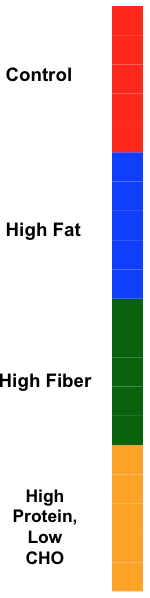

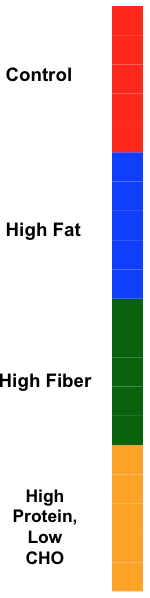


**Fig. S1.** Log fold change of the relative abundance of (A) *L. garvieae* (B) Rosburia and (C) Barnesiellaceae between the first and second fecal sampling for each experimental diet.

| Dataset | All Features LOO Accuracy (%) | Top *X* LOO Accuracy (%) |
| --- | --- | --- |
| Metagenomics (OTU) | 70% | 100% (using 9 features) |
| Brain Regions | 75% | 95% (using 9 features) |

**Table S1.** Accuracy predicting diets using metagenomics and brain region datasets.

|  |  | **Predicted** | | | |
| --- | --- | --- | --- | --- | --- |
|  |  | Diet A | Diet B | Diet C | Diet D |
| **Actual** | Diet A | 2 | 1 | 1 | 1 |
|  | Diet B | 1 | 3 | 1 | 0 |
|  | Diet C | 1 | 0 | 4 | 0 |
|  | Diet D | 0 | 0 | 0 | 5 |

**Table S2.** Leave-One-Out Confusion Matrix (metagenome predicting diet using all OTUs). Better accuracy is achieved after feature selection.

|  |  | **Predicted** | | | |
| --- | --- | --- | --- | --- | --- |
|  |  | Diet A | Diet B | Diet C | Diet D |
| **Actual** | Diet A | 4 | 1 | 0 | 0 |
|  | Diet B | 0 | 4 | 0 | 1 |
|  | Diet C | 0 | 0 | 4 | 1 |
|  | Diet D | 0 | 1 | 1 | 3 |

**Table S3.** Leave-One-Out Confusion Matrix (Brain imaging data predicting diet using ***all*** brain regions).

|  |  | **Predicted** | | | |
| --- | --- | --- | --- | --- | --- |
|  |  | Diet A | Diet B | Diet C | Diet D |
| **Actual** | Diet A | 5 | 0 | 0 | 0 |
|  | Diet B | 0 | 5 | 0 | 0 |
|  | Diet C | 0 | 0 | 4 | 1 |
|  | Diet D | 0 | 0 | 0 | 5 |

**Table S4.** Leave-One-Out Confusion Matrix (Brain imaging data predicting diet using ***selected*** brain regions).

| **Threshold** | **Meta.accuracy** | **Brain.accuracy** | **Combined.accuracy** |
| --- | --- | --- | --- |
| 0.75 | 0.9 | 0.85 | 0.9 |
| 0.76 | 0.9 | 0.85 | 0.9 |
| 0.77 | 0.9 | 0.9 | 0.9 |
| 0.78 | 0.9 | 0.9 | 0.9 |
| 0.79 | 0.95 | 0.9 | 0.9 |
| 0.8 | 0.9 | 0.9 | 0.9 |
| 0.81 | 0.9 | 0.9 | 0.9 |
| 0.82 | 0.9 | 0.9 | 0.9 |
| 0.83 | 0.95 | 0.95 | 0.95 |
| 0.84 | 0.95 | 0.9 | 1 |
| 0.85 | 0.85 | 0.9 | 0.95 |
| 0.86 | 0.8 | 0.9 | 0.95 |
| 0.87 | 0.8 | 0.85 | 0.8 |
| 0.88 | 0.65 | 0.75 | 0.75 |
| 0.89 | 0.65 | 0.75 | 0.75 |
| 0.9 | 0.6 | 0.75 | 0.75 |

**Table S5.** Threshold is the combined score, S_Combined_(OTU_i_,BrainM_j_), and *meta.accuracy*, *brain.accuracy*, and *combined.accuracy* denotes the accuracies of using metagenomic features alone, brain regions alone, and the combined metagenomic and brain region features, respectively.
